# Stress Tolerance of Yeasts Dominating Reverse Osmosis Membranes for Whey Water Treatment

**DOI:** 10.3389/fmicb.2020.00816

**Published:** 2020-05-05

**Authors:** Eirini Vitzilaiou, Stina D. Aunsbjerg, N. A. Mahyudin, Susanne Knøchel

**Affiliations:** ^1^Laboratory of Microbiology and Fermentation, Department of Food Science, University of Copenhagen, Copenhagen, Denmark; ^2^Department of Food Service and Management, Faculty of Food Science and Technology, Universiti Putra Malaysia, Serdang, Malaysia

**Keywords:** filamentous yeast, biofilm, CIP, reverse osmosis, heat tolerance, UV tolerance

## Abstract

Filamentous yeast species belonging to the closely related *Saprochaete clavata* and *Magnusiomyces spicifer* were recently found to dominate biofilm communities on the retentate and permeate surface of Reverse Osmosis (RO) membranes used in a whey water treatment system after CIP (Cleaning-In-Place). Microscopy revealed that the two filamentous yeast species can cover extensive areas due to their large cell size and long hyphae formation. Representative strains from these species were here further characterized and displayed similar physiological and biochemical characteristics. Both strains tested were able to grow in twice RO-filtrated permeate water and metabolize the urea present. Little is known about the survival characteristics of these strains. Here, their tolerance toward heat (60, 70, and 80°C) and Ultraviolet light (UV-C) treatment at 255 nm using UV-LED was assessed as well as their ability to form biofilm and withstand cleaning associated stress. According to the heat tolerance experiments, the D_60_°C of *S. clavata* and *M. spicifer* is 16.37 min and 7.24 min, respectively, while a reduction of 3.5 to >4.5 log (CFU/mL) was ensured within 5 min at 70°C. UV-C light at a dose level 10 mJ/cm^2^ had little effect, while doses of 40 mJ/cm^2^ and upward ensured a ≥4log reduction in a static laboratory scale set-up. The biofilm forming potential of one filamentous yeast and one budding yeast, *Sporopachydermia lactativora*, both isolated from the same biofilm, was compared in assays employing flat-bottomed polystyrene microwells and peg lids, respectively. In these systems, employing both nutrient rich as well as nutrient poor media, only the filamentous yeast was able to create biofilm. However, on RO membrane coupons in static systems, both the budding yeast and a filamentous yeast were capable of forming single strain biofilms and when these coupons were exposed to different simulations of CIP treatments both the filamentous and budding yeast survived these. The dominance of these yeasts in some filter systems tested, their capacity to adhere and their tolerance toward relevant stresses as demonstrated here, suggest that these slow growing yeasts are well suited to initiate microbial biofouling on surfaces in low nutrient environments.

## Introduction

Filamentous yeasts are emerging as highly potent biofilm-forming microorganisms in water distribution systems (Doggett, 2000; Babič et al., 2017), residential dishwashers (Zalar et al., 2011; Döğen et al., 2013; Gümral et al., 2016; Zupančič et al., 2016) and in food industrial equipment (Tang et al., 2009; Tarifa et al., 2013; Stoica et al., 2018; Vitzilaiou et al., 2019). These findings indicate that filamentous yeasts can disperse efficiently, attach strongly to different surfaces and create robust hyphal networks capable of surviving several stresses. According to the study of Paramonova et al. (2009), an increase in hyphae content will strengthen the fungal biofilm and the resistance to such stresses as compression, vortexing and sonication. It has also been shown that filamentous yeasts can develop synergistic relationships with bacteria, leading to vigorous polymicrobial biofilm structures (Shirtliff et al., 2009; De Brucker et al., 2015; Zupančič et al., 2018).

Spiral wound RO membranes are widely used in the food industry and the recovered watery permeate may be used for different purposes. The membranes offer high filtration efficiency, but they are susceptible to biofouling causing flux reduction and shortening of membrane life (Herzberg and Elimelech, 2007; Tang et al., 2009; Anand et al., 2012, 2013; Stoica et al., 2018). Biofouling may also become an issue for final product quality, if microbial cells from the biofilms are capable of proliferating further down the processing line or in the water permeate during storage, since the water may be used for cleaning or for direct/indirect product contact processes (Casani and Knøchel, 2002; Casani et al., 2005).

Over the last decades, there has been an increasing interest in spiral wound RO membrane biofouling in the food processing industry (Tang et al., 2009; Hassan et al., 2010; Anand et al., 2012, 2013; Sánchez, 2018; Stoica et al., 2018; Vitzilaiou et al., 2019). Up to now, research has been mainly focused on bacterial contamination. In 2009, however, a filamentous yeast isolate was observed among the bacterial isolates on RO membranes from a dairy industry in New Zealand. It was isolated from the retentate side of RO elements used for processing casein water permeate and identified as the filamentous yeast *Blastoschizomyces capitatus* (Tang et al., 2009), later renamed *Magnusiomyces capitatus* (Sybren De Hoog and Smith, 2011a).

We have recently documented that filamentous yeasts belonging to the closely related species *Saprochaete clavata* and *Magnusiomyces spicifer* may dominate biofilms found after CIP treatment on both sides of RO membranes filtering whey water (Stoica et al., 2018; Vitzilaiou et al., 2019). Although these biofilms may have a great impact on RO filtration efficiency, little is known about these isolates and their response to the stress encountered. Here, our aim is to characterize two representative strains of these filamentous yeasts (*S. clavata* and *M. spicifer*) isolated from RO elements (Stoica et al., 2018; Vitzilaiou et al., 2019) with focus on properties of importance for survival and growth in the processing environment. Morphological and physiological–biochemical tests were conducted and the heat and UV-C (255 nm) light tolerance of the filamentous yeasts was investigated. The ability of one of the filamentous yeasts to create biofilms on polystyrene flat-bottomed microwells and on peg lids, respectively, was assessed and compared to that of a budding yeast strain, *Sporopachydermia lactativora*, found on the same biofilm structures. The tolerance of biofilms on RO membrane coupons was tested toward different industrial CIP-mimicking treatments in lab-scale experiments to broaden our knowledge about these RO membrane biofouling agents and facilitate future development of targeted cleaning operations.

## Materials and Methods

### Yeast Strains’ Isolation

The filamentous yeast strains belonging to *S. clavata* and *M. spicifer* and the budding yeast strain belonging to *S. lactativora*, investigated here, are representative strains from the total number of yeast species previously isolated from a RO membrane filtration line for water reuse in a dairy industry (Stoica et al., 2018; Vitzilaiou et al., 2019). *S. clavata* and *M. spicifer* found to be the dominant yeast species and *S. lactativora* the most commonly found budding yeast in the detected biofilm structures. In the set up investigated, the whey was up-concentrated through Ultrafiltration (UF) and the permeate solution further subjected to two consecutive RO filtrations and two UV-C (Ultraviolet) light steps before reuse. Sampling, isolation and sequencing procedures are described in Stoica et al. (2018) and Vitzilaiou et al. (2019).

### Macroscopic and Microscopic Characterization

Phase-contrast microscopy using the upright microscope Olympus BX43 (Olympus Scientific Solutions Americas Corp.) was used to observe the yeast cells grown in YPG broth (20 g/L Glucose, 20 g/L Peptone, 10 g/L Yeast Extract, pH 5.6 ± 0.2) at 25°C with shaking at 225 rpm (orbital shaker: IKA KS 130 control) and Malt Extract Agar (MEA) at 25°C (CM0059/Oxoid).

### Strains Used for Physiological–Biochemical Tests

An isolate from each of the two dominant filamentous yeast species was selected for physiological–biochemical tests. These isolates will be referred to as *SC, for Saprochaete clavata* and *MS, for Magnusiomyces spicifer*, throughout the rest of the paper. Growth at different temperatures (5, 25, 30, 35, 37, 40, and 45°C) without shaking was assessed in 10 mL YPG broth tubes, inoculated with an individual colony (MEA agar plates/25°C) with triplicates for each isolate and temperature. Fermentation of carbohydrates (D-glucose, D-galactose, sucrose, maltose, lactose, raffinose, and trehalose) and assimilation of carbon compounds (D-glucose, D-galactose, sucrose, maltose, lactose, raffinose, trehalose, D-xylose, L-sorbose, cellobiose, salicin, DL-lactate, succinate, citrate) and nitrate were tested in tubes, in triplicates, incubated at 25°C without shaking, according to the experimental procedure of Kurtzman et al. (2011). Growth at different temperatures and results of fermentation and assimilation experiments were assessed after 1, 2, 3, and 4 weeks of incubation, respectively, using the Wickerham card. Urease activity was tested using Christensen’s urea broth (Kurtzman et al., 2011).

### Growth in Twice RO-Filtrated Water

The ability of *SC* and *MS* to grow in the twice RO-filtrated permeate water was assessed. One individual colony from *SC* and *MS*, respectively, was inoculated in tubes containing 10 mL of twice RO-filtrated permeate water from the same line (stored in the fridge/4°C). After inoculation, the tubes were incubated for 3 days at 25°C with shaking at 225 rpm. These tubes and the non-inoculated control sample (twice RO-filtrated permeate water) were analyzed to determine the urea and ammonia concentration using an enzyme assay as described by the manufacturer (Megazymes, United Kingdom).

### Heat Tolerance Assay

Planktonic cell suspensions of *SC* and *MS* in Saline Peptone Solution (SPS) [1 g/L Bacto Peptone (Difco 211677), 8.5 g/L sodium chloride, pH 7.2 ± 0.2] were exposed to 60, 70, and 80°C for 5, 10, 15, and 20 min, and the CFU/mL determined on MYPG agar (10 g/L glucose, 5 g/L peptone, 3 g/L yeast extract, 3 g/L malt extract, 20 g/L agar, pH 5.6 ± 0.1) in order to assess the heat tolerance. For the inocula, the two filamentous yeast isolates (*SC* and *MS*) were incubated separately on MYPG agar plates at 25°C for 3 days. For each of them, material from an individual colony was inoculated into 20 mL YPG broth tubes and incubated with shaking at 25°C for 2 days. Cells were harvested from the liquid cultures by centrifugation (3,000 g/5 min/4°C). The supernatant was removed, cold (4°C) SPS added, and the pellet washed by vortexing, followed by centrifugation. This procedure was repeated twice, and the pellet was re-suspended in 20 mL SPS and vortexed. The Neubauer counting chamber was used to create a 10^7^ cells/mL inoculum in SPS. For each temperature-time interval, 1.5 mL sterile eppendorf tubes with 990 μL SPS were inserted in the pre-heated heating blocks and the temperature monitored in a control tube. When the solution reached the desired temperature, 10 μL from the initial inoculum was added to the eppendorf tubes for a final concentration of 10^5^ cells/mL. Triplicates were made for each isolate and temperature-time interval. After each designated temperature-time interval, tubes were placed in ice and serial dilutions made in SPS followed by spread plating on MYPG agar plates. Initial inocula were also validated on MYPG agar. Colonies were counted after 10 days of incubation at 25°C, to allow for growth of injured cells. *D*-values for 60°C for *SC* and *MS* were calculated using linear regression analysis (Murphy et al., 2000, 2002; McCormick et al., 2003).

### UV-C Light Treatment

UV-C light treatment at 254 nm of 40 mJ/cm^2^ is commonly applied to RO permeate water in the finishing steps before storage and reuse. The tolerance of the two filamentous yeast isolates to UV-C light treatment was investigated, using PearlLab Beam Device from AquiSense Technologies (Kentucky, United States), a compact Collimated Beam Device employing UV-LEDs and emitting UV-C irradiation at 255 nm, following the Bolton and Linden (2003) and Bolton et al. (2015) protocols. The inoculum was prepared as described in Section “Heat Tolerance Assay” and the inoculum concentration set at 10^6^ cells/mL. The (%) UV transmittance of the SPS microbial suspension at 255 nm (UV-Vis 1800 spectrophotometer, Shimadzu) was measured to calculate the water factor. The center point irradiance [*E*_*o*_, (mW/cm^2^)] of the UV-C LED device was determined (radiometer ILT2400, International Light Technologies) and used together with the water factor, petri factor, divergence factor, and sensor factor to calculate the average germicidal fluence rate (*E*′_*avg*_). To obtain the exposure time (sec) for a desired UV dose, this dose (mJ/cm^2^) was divided by the average fluence rate (mW/cm^2^). The selected UV doses were 10, 20, 40, 60, and 100 mJ/cm^2^. The exposure time (sec) needed to achieve the desired UV-doses was calculated according to the protocol. Four individual trials were conducted with two replicates within each trial. For each UV dose, 20 mL inoculum (4°C) were transferred to a 50 mm sterile petri dish and exposed to UV-C light for specific time (sec) to achieve the desired dose. The cell suspensions were continuously stirred during the experiments. UV doses were applied in a random order. For each trial, there was a control UV-untreated sample. UV treated and untreated samples were spread-plated in MYPG agar and incubated up to 10 days at 25°C. CFU/mL were counted and survival graphs were made. Two Sample Welch *t*-test was applied with a significance level of 0.05 (*P* < 0.05), using R studio software (Version 3. 6. 1), to assess the differences between the two strains’ inactivation profile for the same UV dose. One-way analysis of variance (ANOVA) and *post hoc* Tukey test with a significance level of 0.001 (*P* < 0.001) were conducted to assess the differences among the different doses in microbial population reduction for both strains, using R studio software (Version 3. 6. 1).

### Cleaning-In-Place (CIP) Tolerance Assay

**FIGURE 1:**
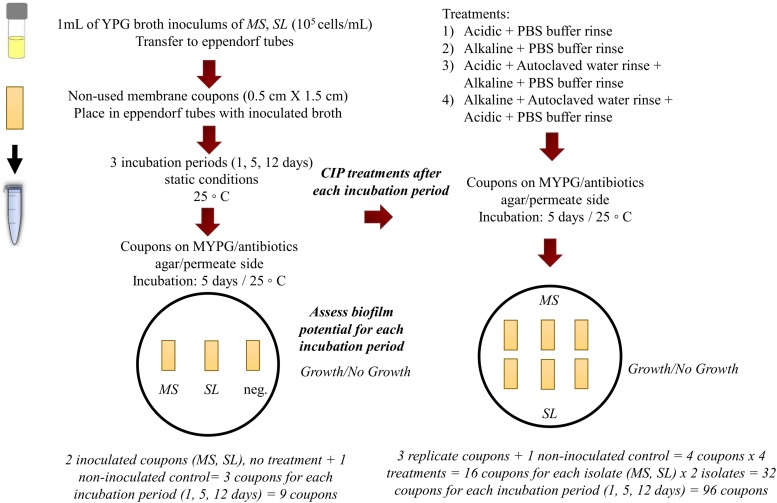
Experimental design of the CIP tolerance assay.

The tolerance of the filamentous yeast strain *MS* toward CIP treatments was compared to that of the budding yeast strain, *S. lactativora*, *SL*, both isolated from the RO membrane biofilms, by forming biofilms of single cultures on RO membrane coupons and exposing them to the different industrial CIP treatments on a lab-scale experiment. The coupons of 0.5 × 1.5 cm were aseptically cut from an unused RO membrane, which had been stored at 4°C in sodium metabisulfite solution (1.0% w/w). The coupons were flushed by pipetting three times from each side with autoclaved water to remove chemical traces and placed into sterile 1.5 mL eppendorf tubes. Inocula of 10^5^ cells/mL were prepared for each strain with YPG broth as the suspension solution (see section “Heat Tolerance Assay”). For each isolate, 1 mL of the inoculum was poured into a tube, covering the coupon, and incubated for 1, 5, or 12 days at 25°C. For each incubation period, a positive (inoculated) and a negative control (non-inoculated) were made for the two isolates (*MS* and *SL*) (Figure 1). In order to mimic a CIP program, the operating cleaning agents and time-temperature combinations from industrial CIP programs were used (Figure 1 and Table 1). The experiment consisted of four different treatments. The first involved only the application of the acidic solution and the second only the alkaline. The third treatment applied the acidic solution before the alkaline, while the fourth applied the alkaline first, followed by the acidic. In the third and fourth treatment, there was a rinsing step by pipetting 1 mL of autoclaved water from each side of each coupon three times between the acidic and alkaline solution. At the end of all the treatments, there was a rinsing step by pipetting 1 mL of Phosphate Buffer Solution from each side of the coupon three times in a standardized manner before placing on agar (PBS: 8.00 g/L NaCl, 0.20 g/L KCl, 1.44 g/L Na_2_HPO_4_ and 0.24 g/L of KH_2_PO_4_, pH 7.4 ± 0.2). The CIP solutions were prepared according to the protocol of the producer using autoclaved water for dilution (Figure 1 and Table 1). For each treatment, three replicate coupons in inoculated YPG broth and one coupon in non-inoculated YPG broth (control) were made for each isolate (*MS* and *SL*) and for each incubation period (1, 5, and 12 days) (Figure 1).

**TABLE 1:** The four different CIP treatments applied in the lab-scale experiment for the filamentous and budding yeast biofilm removal.

**CIP treatments**				
1	Acidic cleaning solution (nitric acid and citric acid)		PBS buffer rinse	
	45 min/50°C			
	pH 1.8–2.0			
2	Alkaline cleaning solution (potassium hydroxide, EDTA, and sodium hydroxide)		PBS buffer rinse	
	35 min/50°C			
	pH 11.0–11.5			
3	Acidic cleaning solution (nitric acid and citric acid) 45 min/50°C pH 1.8–2.0	Autoclaved water rinse	Alkaline cleaning solution (potassium hydroxide, EDTA, and sodium hydroxide) 35 min/50°C pH 11.0–11.5	PBS buffer rinse
4	Alkaline cleaning solution (potassium hydroxide, EDTA, and sodium hydroxide) 35 min/50°C pH 11.0–11.5	Autoclaved water rinse	Acidic cleaning solution (nitric acid and citric acid) 45 min/50°C pH 1.8–2.0	PBS buffer rinse

The biofilm formation on the coupons was assessed by placing the inoculated coupons from each incubation period and isolate on MYPG/antibiotics agar along with the non-inoculated control with the permeate side facing downward. For the CIP tolerance assay, the triplicate coupons tested for each incubation period and isolate combination as well as the non-inoculated controls were exposed to the four different treatments (Figure 1). Eppendorf tubes were filled with 1 mL of the appropriate CIP solutions, corresponding to the treatment, and pre-heated to 50°C in heating blocks to mimic the temperature of the cleaning solutions during industrial CIP. The incubated RO membrane coupons were placed in these tubes and exposed to the various treatments. After each treatment the coupons were placed on MYPG/antibiotics agar supplemented with 0.1 g/L chloramphenicol (Sigma C0378) and 0.5 g/L chlortetracycline (Sigma C4881) with the permeate side facing downward. For both the biofilm forming potential and the CIP tolerance assays, the coupons were removed from the agar after 1 day and the plates were further incubated at 25°C for 5 days. Growth/no growth was macroscopically assessed on the agar plates after 5 days of incubation (Figure 1 and Table 1).

### Biofilm Formation on Polystyrene Microtiter Plates and Peg Lids

Biofilm formation on flat-bottomed polystyrene microtiter plates (Thermo Scientific^TM^ Nunc^TM^ MicroWell^TM^) was assessed using 0.1% Crystal Violet (CV) staining, indicative of biomass level, and tetrazolium salt (XTT) (Sigma, cat.no.X4251) staining with 1 μM menadione (Sigma, cat.no.M5625), indicative of metabolic activity. Inocula of the filamentous yeast *SC* and the budding yeast *SL* were prepared (10^5^ cells/mL) as in Section “Heat Tolerance Assay.” Three different broth media were used for the final suspension: a low nutrient broth (R2B) containing 0.5 g/L yeast extract, 0.5 g/L proteose peptone, 0.5 g/L casein hydrolysate, 0.5 g/L glucose, 0.5 g/L starch, 0.5 g/L di-potassium phosphate, 0.024 g/L magnesium sulfate, 0.3 g/L sodium pyruvate (pH 7.2 ± 0.2); R2B + with 100 mg/L urea and lactose, respectively (approx. concentrations found in the twice filtered RO membrane permeate samples), and a high nutrient broth, YPG. Three incubation periods were applied: 1, 4, and 7 days. For each isolate, incubation period and staining method, a microtiter plate was inoculated with the three different media (200 μL/triplicate rows/eight wells). After 1, 4, and 7 days of incubation with shaking (25°C, 140 rpm), broth media were aspirated from the microplates, where after the microplates were washed (200 μL PBS/three times) and stained with 0.1% CV [protocol of Kirchhoff et al. (2017)] and 1 μM XTT/menadione [protocol of Pierce et al. (2008)]. For the 0.1% CV assay, 125 μL of 0.1% CV solution were poured into each well and the microplates were incubated for 20 min in room temperature (RT). After three additional washing steps with 200 μL PBS, the microplates were air-dried in inverted position at room temperature (RT) (22°C) for 3 h and 200 μL 30% (v/v) acetic acid were added in each well. After 30 min incubation at RT, 150 μL from each well were transferred to new microplates for each yeast strain and OD was measured at 600 nm. For the XTT/menadione assay, 100 μL of the prepared 1 μM XTT/menadione solution were added to the microplates after draining. After incubation for 3 h at 25°C/dark conditions, 80 μL from each well were transferred to new microplates for each yeast strain and the OD was measured at 490 nm. OD was measured using the microtiter plate reader BioTeck ELx808. For both assays, the average OD value for each of the inoculated media wells was calculated by subtraction of the average OD value from the non-inoculated media wells. Student’s paired *t*-test was conducted with a significance level of 0.001 (*P* < 0.001) to assess the differences for each medium between the different incubation periods. R studio software (Version 3. 6. 1) was used for the statistical analysis.

Biofilm formation was also assessed on peg lids (MBEC^TM^ P&G Assay/Innovotech) for both single and dual *SC* and *SL* yeast suspensions, applying three sonication times (10, 15, and 20 min). The method was adapted from Harrison et al. (2010). Inocula of 10^5^ cells/mL were prepared for the single yeast and dual yeast biofilms, using YPG broth for the final suspension (see section “Heat Tolerance Assay”). For each sonication time: a microplate was prepared for the single yeast biofilm by inoculation of columns 1–6 with 150 μL of *SC* inoculum and of columns 7–12 with 150 μL of *SL* inoculum (48 wells for each strain). For the dual yeast biofilm, 75 μL of *SC* and *SL* inocula, respectively, were added to a microplate (final volume: 150 μL, columns 1–12, 96 wells). The peg lids were inserted into the two inoculated microplates and incubated for 48 h at 25°C with shaking (140 rpm). After incubation, the peg lids were removed from the single and dual yeast microplates and rinsed by submerging subsequently for 1 min in two microplates containing Rinse Solution (PBS: 200 μL/well), then submerged into microplates containing Recovery Solution (YPG broth: 200 μL/well) and sonicated for 10 min (sonicator: Branson 2210R-MT Ultrasonic Cleaner, frequency: 40 kHz). The same procedure was repeated for the 15- and 20- min sonication time. After each sonication time, 20 μL from 12 wells of the single *SC* sonicated microplate were diluted into the first row of a microplate for dilutions, containing 180 μL of Recovery solution. Four dilutions were made in total (A-D/columns 1–12). Control wells were also prepared, using non-inoculated YPG broth. 10 μL from each well (rows A–D) and from the control wells were spotted on YPG agar. The same procedure was repeated for *SL* and for the dual biofilm microplate. The YPG agar plates were incubated at 25°C for 2–3 days. Log_10_ (CFU/mL) was calculated for the single and dual biofilms for the three different sonication times, by calculating the average of 12 wells from the colonies of countable dilutions. Student’s paired *t*-test was conducted to assess individually the differences for the *SC* single and the dual *SC&SL* biofilm cultures for the different sonication times with a significance level of 0.001 (*P* < 0.001). Two Sample Welch *t*-test was applied to assess the differences between *SC* single and the dual *SC&SL* biofilm cultures for the same sonication time with a significance level of 0.001 (*P* < 0.001). R studio software (Version 3. 6. 1) was used for the statistical analysis.

## Results

### Macroscopic and Microscopic Analysis of the Filamentous Yeast Isolates

**FIGURE 2:**
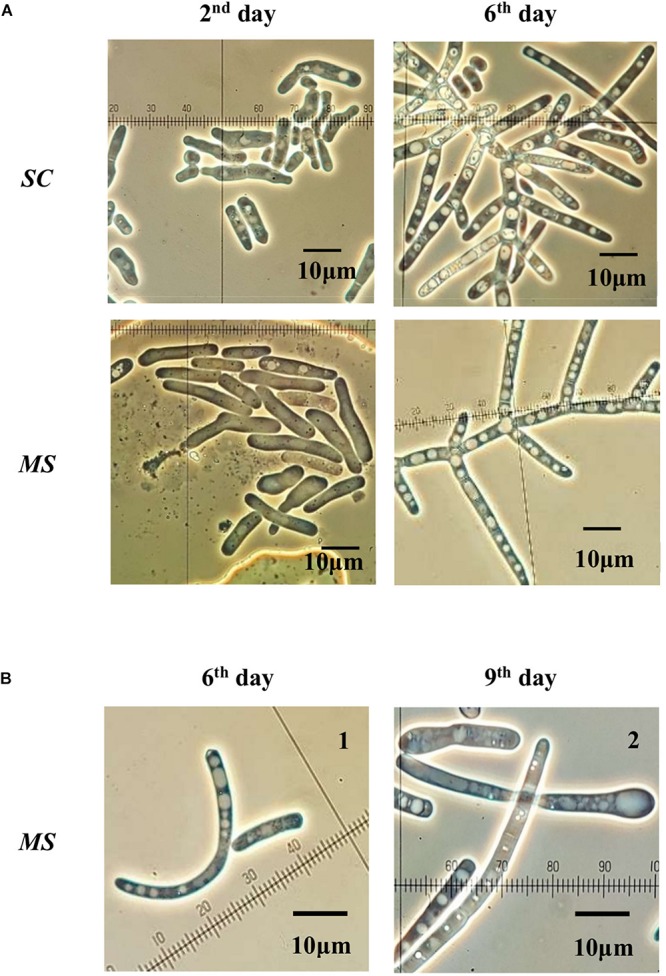
**(A)** *Saprochaete clavata* (*SC*) and *Magnusiomyces spicifer* (*MS*) cells incubated in YPG broth for 2 and 6 days. **(B)** Characteristic cell structures found in *MS* isolate. 1: semi-circle cell structure observed after 6 days in YPG broth. 2: Swollen cells at the apex of the filament, observed after 9 days in YPG broth. Incubation at 25°C with shaking at 225 rpm.

On MEA agar, after 12 days incubation at 25°C, *SC* colonies were circular with a 2–7 mm diameter, glassy, tough, hirsute, white, convex, and filiform with 1–4 mm mycelium length, while *MS* colonies were circular with a 1–3 mm diameter, butyrous, glistening, soft, whitish, convex, and filiform with 1 mm mycelium. Overall, *MS* developed a much shorter mycelium than *SC*. On MEA agar, after 4 days of incubation at 25°C, the budding yeast *SL* strain formed circular, glistening, butyrous, creamy colonies without mycelium and approx. 2 mm in diameter (Table 2). Microscopically, *SC* and *MS* developed septate branching hyphae that elongated by continuous growth of the hyphal tip followed by formation of septa after 6 days of incubation in YPG broth. Septa were refractive and thick with little or no constriction and arthroconidia were rectangular-rounded. They both had large vacuoles, while *MS* developed swollen terminal cells or semi-circle cells (Figure 2). Cell size was measured for at least 20 cells for each yeast strain in the microscope and the minimum-maximum values were 10 and 350 μm in length and 3 and 7.5 μm in width. *SL* cells were ovoid-ellipsoid with minimum-maximum cell values of 4 and 6 μm in length and 2 and 4 μm in width. Multilateral budding on a narrow base was observed for *SL* (Table 2).

**TABLE 2:** The selected strains for the different physiological–biochemical tests and stress tolerance assays.

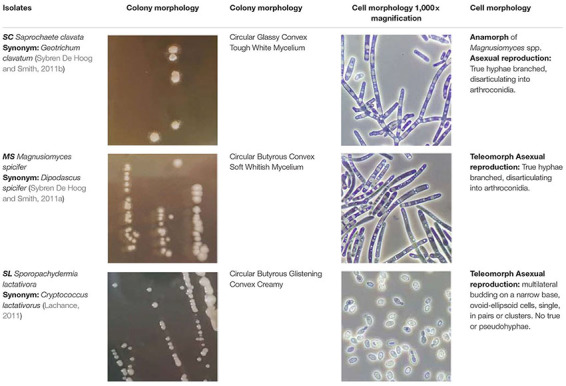

*S. clavata and *M. spicifer*: colonies in MEA agar/12 days incubation/25°C, cells in YPG broth/12 days incubation/25°C/shaking at 225 rpm, Sporopachydermia lactativora: colonies and cells in MEA agar/4 days/25°C.*

### Physiological and Biochemical Tests

Both *SC* and *MS* grew at all temperatures tested in YPG broth. They could not ferment the sugars tested (glucose, sucrose, lactose, galactose, raffinose, trehalose, and maltose), but they assimilated several carbon compounds, corresponding to the species’ definition of Sybren De Hoog and Smith (2011b) for *S. clavata* and of Sybren De Hoog and Smith (2011a) for *M. spicifer*. Christensen’s urea broth reaction for urease activity was negative for both isolates (Table 3).

**TABLE 3:** Assimilation of carbon compounds and nitrate*, urease activity** and sugar fermentation*** for *S. clavata* and *M. spicifer*.

	**Assimilation of carbon compounds**				**Sugar fermentation**				
**Strains**	***S. clavata* (This study)**	***S. clavata* (Sybren De Hoog and Smith, 2011b)**	***M. spicifer* (This study)**	***M. spicifer* (Sybren De Hoog and Smith, 2011a)**	***S. clavata* (This study)**	***S. clavata* (Sybren De Hoog and Smith, 2011b)**	***M. spicifer* (This study)**	***M. spicifer* (Sybren De Hoog and Smith, 2011a)**	***S. clavata* (This study)**
2-5Glucose	+	+	+	+	−	−	−	−	−
Sucrose	−	−	−	−	−	−	−	−	−
Lactose	−	−	−	−	−	−	−	−	−
Galactose	+	+	+	+	−	−	−	−	−
Raffinose	−	−	−	−	−	−	−	−	−
Trehalose	−	−	−	−	−	−	−	−	−
Maltose	−	−	−	−	−	−	−	−	−
D-Xylose	Weak	−	+	+					
L-Sorbose	Slow	+	Slow	+					
Cellobiose	+	+	Slow	+					
Salicin	+	+	Slow	+					
DL-Lactate	Weak	+/Weak	Weak	+					
Succinate	Weak	+	Weak	+					
Citrate	Weak	+	Weak	+					
Nitrate	−	−	−	−					
Urease activity (Christensen’s urea broth)	−	−	−	−					

Triplicates were made for each compound and strain (n = 3) *Tubes were assessed after 1, 2, 3, and 4 weeks with the Wickerham card. +: growth after 1–2 weeks. Slow: growth after 3 weeks. Weak: growth after 4 weeks. −: no growth during incubation. **Although negative for urease activity, the strains may metabolize urea through other pathways. ***Inverted Durham tubes were assessed for gas accumulation after 1, 2, 3, and 4 weeks. −: no accumulation of gas.

### Growth in Twice Filtrated-RO Permeate Water

Although both filamentous yeast isolates were negative for the Christensen’s urea broth reaction, they both grew in the twice RO-filtrated permeate water containing urea and decreased the level of urea by more than 50 mg/L while producing ammonia (Table 4).

**TABLE 4:** Urea reduction and ammonia production in twice RO-filtrated permeate water by growth of the two filamentous species.

	**Control (not inoculated RO water)**	***Saprochaete clavata* in RO water**	***Magnusiomyces spicifer* in RO water**
Urea* (mg/L)	130.7	78.2	66.9
Ammonia* (mg/L)	0.0	24.7	35.2

*Detection limit: ammonia: 2.8 ppm, urea: 5.1 ppm.

### Heat Tolerance Assay

**FIGURE 3:**
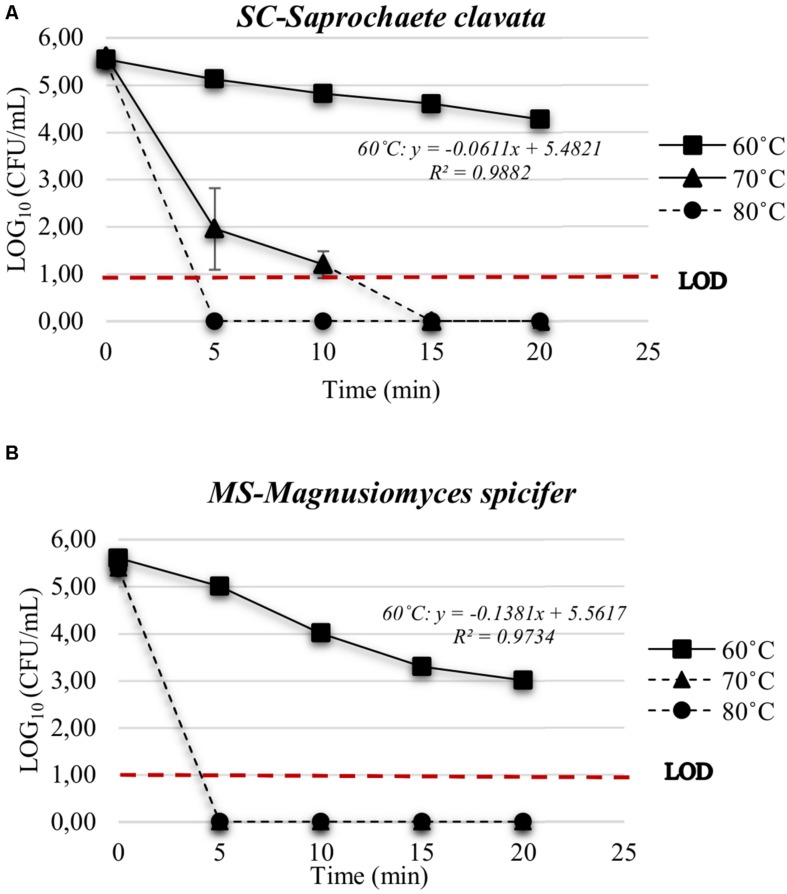
Heat inactivation of **(A)** *S. clavata* (*SC*) and **(B)** *M. spicifer* (*MS*) in SPS at 60, 70, and 80°C for 5, 10, 15, and 20 min. Phantom lines indicate that population level was below the LOD (10 cells/mL). Mean value and standard deviation were calculated from three technical replicates for each temperature-time interval and strain (*n* = 3). Medium: MYPG agar. Incubation: 10 days/25°C.

The initial inoculum for *SC* (*S. clavata*) and *MS* (*M. spicifer*) was approx. 10^5^ cells/mL (Figure 3). The cell suspensions of *SC* and *MS* were tolerant to heat treatment at 60°C, being reduced less than 2 and 3 log_10_ (CFU/mL), respectively, after 20 min *SC* was still detectable after exposure at 70°C for up to 10 min, while *MS* became undetectable after 5 min. At 80°C, *SC* also became undetectable within 5 min. The *D*-value for *SC* at 60°C was *D*_60°*C*_ = *16.37 min*, while the *D*-value of *MS* was *D*_60°*C*_ = *7.24 min.*

### UV-C Light Treatment

**FIGURE 4:**
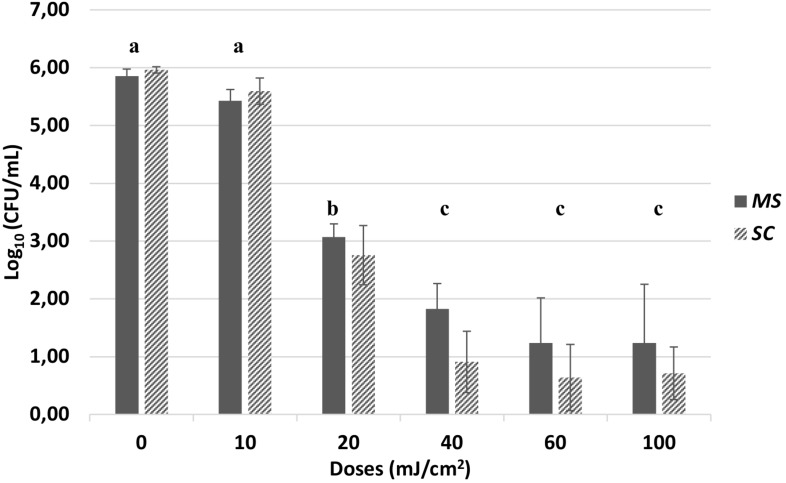
Population level in Log_10_ [CFU/mL of *M. spicifer* (*MS*) and *S. clavata* (*SC*)] after exposure to UV doses of 0, 10, 20, 40, 60, and 100 mJ/cm^2^. Inoculation level: 10^6^ cells/mL. LOD: 1 CFU/mL. The average and standard deviation has been calculated from four different trials with two replicates within each trial (*n* = 8). Sporadic survivors were found when applying 200 and 400 mJ/cm^2^ (Data not shown). No significant differences were found (*P* > 0.05) between the two strains for each dose. The different letters indicate the significant differences in microbial reduction among the different doses for both isolates (*P* < 0.001). Agar medium: MYPG, 10 days incubation at 25°C.

The survival bars in log_10_ (CFU/mL) for *MS* and *SC*, respectively, for the different UV doses are presented in Figure 4. Both isolates exhibited high tolerance to UV-C treatment. Exposure to 10 mJ/cm^2^ resulted in less than 1.00 log_10_ (CFU/mL) and exposure to 20 mJ/cm^2^ in less than 4.00 log_10_ (CFU/mL) reduction. A UV dose of 40 mJ/cm^2^, usually applied in the industrial UV-C water treatment lines, resulted in approx. 4.00 log_10_ (CFU/mL) reduction for *MS* and 5.00 log_10_ (CFU/mL) reduction for *SC*. Exposure to 60 mJ/cm^2^ resulted in approx. 1 log_10_ (CFU/mL) further reduction for both strains. There was no higher decrease in population by applying 100 mJ/cm^2^. After doses of 200 and 400 mJ/cm^2^, applied in two of the trials, sporadic survivors were found (Data not shown). Two Sample Welch *t*-test with a significance level of 0.05 showed that the differences in population level reduction between the two strains for each dose applied were no significant (*P* > 0.05). One-way ANOVA and *post hoc* Tukey test conducted, showed that for both strains, there was significant reduction in microbial population after 20 and 40 mJ/cm^2^ (*P* < 0.001), compared to dose 0. However, there was not significant difference in the reduction among the doses of 40, 60, and 100 mJ/cm^2^ (*P* > 0.001).

### CIP Tolerance Assay

Biofilms developed already after the 1st day on the membrane coupons for both the filamentous (*MS*) and the budding (*SL*) yeast isolate, and later increased further in biomass, as seen in the upper row of Table 5. Exposure to individual and combined treatments with the alkaline and the acidic cleaning solution did not eliminate the biofilms of neither *SL* nor *MS* strain. However, the combined treatments were more effective than the individual ones in biofilm removal, especially after 1 day of coupon incubation while the more mature biofilms were difficult to eradicate. This was especially true for the *SL*. Changing the succession of the acidic and alkaline solution during CIP did not seem to improve biofilm removal.

**TABLE 5:** Tolerance toward different CIP treatments at 50°C of single filamentous and budding yeast biofilms, formed on RO membrane coupons.

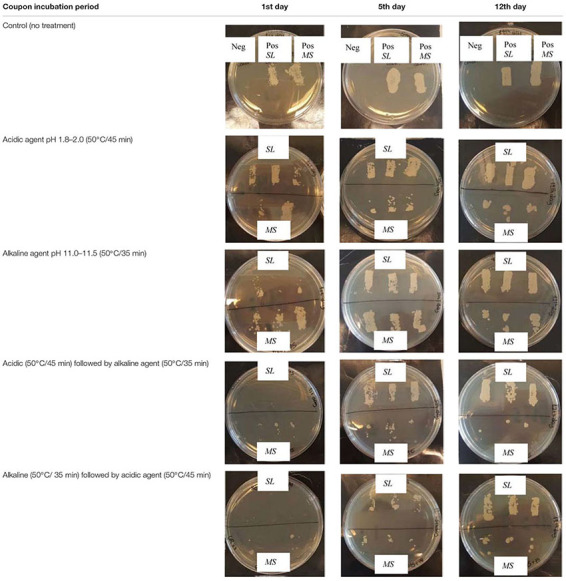

*Neg, negative coupon-control in non-inoculated YPG broth/no treatment. Pos, positive coupon-control in inoculated YPG broth/no treatment. SL, budding yeast (S. lactativora); MS, filamentous yeast (M. spicifer). Triplicates were made for each CIP treatment and strain. Initial inoculum: 10^5^ cells/mL. Agar plates’ incubation: MYPG/antibiotics, 12 days, 25°C. Negative controls for CIP treatments for rows 2–5 not shown.*

### Biofilm Formation on Polystyrene Microplates

**FIGURE 5:**
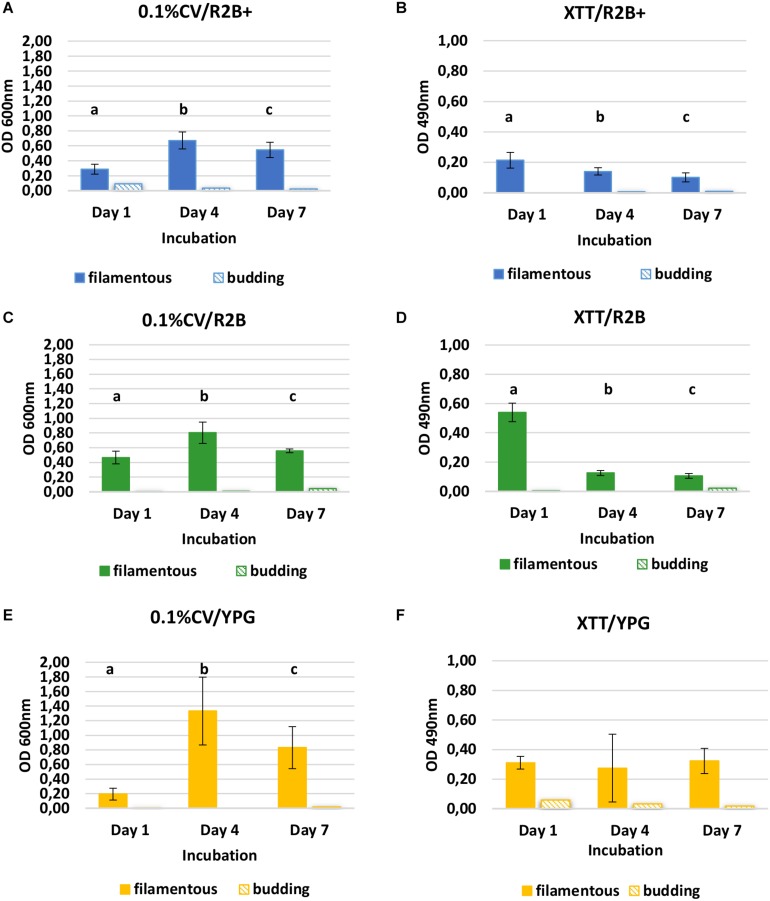
Biofilm quantification of the filamentous yeast *S. clavata* and budding yeast *S. lactativora* using 0.1% CV and XTT/menadione stains in R2B + with urea and lactose **(A,B)**, R2B **(C,D)**, and YPG broth **(E,F)** after 1, 4, and 7 days of incubation (25°C, shaking at 140 rpm). Mean value and standard deviation are calculated from 24 replicate wells for each medium. Significant differences for the filamentous yeast are presented for each graph with the different letters (a, b, c) (*P***<** 0.001).

*Saprochaete clavata* created strong biofilm in the flat-bottomed polystyrene microtiter well assay in all the different media, according to both staining methods used (Figure 5). The biomass (CV) increased significantly from the first to the fourth day of incubation in all the different media (Figures 5A,C,D) (*P* < 0.001), while at the same time the metabolic activity (XTT) decreased significantly in R2B + and R2B (Figures 5B,D) (*P* < 0.001). In XTT/YPG, there were no statistically significant differences found throughout incubation (Figure 5F) (*P* < 0.001). From Days 4 to 7, both biomass (CV) and metabolic activity (XTT) decreased, indicating cell death. *SL* did not attach and create biofilms on polystyrene. OD was between 0.000–0.092 in 0.1% CV and between 0.000–0.058 in XTT/menadione for the different media and incubation days.

The biofilm formation on peg lids also supported that the filamentous *SC* had the ability to form biofilm, while no biofilm formation was detected for the tested budding yeast *SL* (Table 6). The filamentous yeast *SC* biofilm had high recovery numbers for all tested sonication times, while *SL* was below the detection limit of the method (100 cells/mL). The biofilm recovery numbers were statistically significantly lower in the dual *SC&SL* biofilm compared to the single *SC* biofilm for 10 and 15 min (*P* < 0.001). However, no differences were observed between the single and the dual biofilm after 20 min (*P* > 0.001). Regarding the different sonication times, 15 min were more efficient in biofilm cell recovery numbers. The decrease in cell recovery numbers after 20 min could be due to inactivation of the yeast after prolonged sonication at 40 kHz.

**TABLE 6:** Biofilm cell counts on peg lids obtained from Recovery Solution (YPG) at 10-, 15-, and 20-min sonication time for *S. clavata* (*SC*), *S. lactativora* (*SL*) single biofilms, and *SC/SL* dual biofilms.

**Sonication time (min)**	**10**	**15**	**20**
	
	**Log_10_ (CFU/ml)**		
*SC* (Single biofilm)	4.73 ± 0.17^a^	5.25 ± 0.22^b^	4.16 ± 0.20^c^
*SL* (Single biofilm)	<LOD	<LOD	<LOD
*SC*&*SL* (Dual biofilm)	4.33 ± 0.11^d^	4.45 ± 0.12^d^	3.90 ± 0.34^c^

*Data from control wells was negative (not shown). Mean values and standard deviation were calculated from 12 replicates. Significant differences within SC and SC&SL biofilms for the different sonication times and between SC and SC&SL biofilms for the same sonication time are indicated with different letters (a, b, c, d) (P < 0.001). LOD was 100 cells/mL.*

## Discussion

### Isolation and Characterization of the Filamentous Yeast Isolates

The filamentous yeast species *S. clavata* and *M. spicifer* dominated the biofilm communities on the retentate and permeate side of RO membranes used in a dairy operation line for treating whey water. These filamentous yeasts were isolated after CIP application on several occasions from elements having been in use for 6 months and up to 4 years. Interestingly, the biofilms on the permeate side of most of these elements, consisted exclusively of the filamentous yeast strains. Since biofouling can interfere with flux and may potentially affect the quality of the retentate and permeate, there is clearly an interest in characterizing these yeasts and obtain an understanding of their persistence and role. It has already been established that they may cover large areas and still go relatively unnoticed unless selective media are used for their detection, since they are often present in lower numbers and grow slower than bacteria, causing them to be out-grown on non-selective media (Stoica et al., 2018; Vitzilaiou et al., 2019).

The yeasts *S. clavata* and *M. spicifer* have a high degree of similarity in physiology, cell-colony morphology and biochemical profile. They both developed mycelium on agar after long incubation and their cells elongated and created septate branching hyphae. They had the same temperature growth range and assimilated the same carbon compounds, while they did not assimilate nitrate. According to taxonomy, they are closely related species. *Saprochaete* and *Magnusiomyces* genera belong to the same phylum, class and order (Ascomycota, Saccharomycetes, and Saccharomycetales). *Saprochaete* is the anamorph of *Magnusiomyces* and *Magnusiomyces* and *Saprochaete* are sister genera to *Galactomyces, Dipodascus* and their anamorph *Geotrichum* (Sybren De Hoog and Smith, 2004, 2011a,b). The differentiation between *Saprochaete* and *Magnusiomyces* is difficult as also reported by Del Principe et al. (2016). The identification results in the NCBI Database, based on 26S and ITS rRNA sequencing, were often giving similar matches in terms of (%) coverage, (%) similarity rates and *E*-values for *M. spicifer* and *S. clavata*. Thus, we believe that there is a need to increase the diversification of the existing databases on fungal species, as well as to develop new methods for fungal species identification.

### Urea Metabolism

Ultrafiltration followed by RO membrane filtration will, in theory, remove the fouling agents and microbial cells, while small molecules may pass through the membrane pores. Urea from whey can pass the RO membranes (Skou et al., 2017, 2018) and may therefore be a source of nitrogen and energy for some microorganisms in the low-nutrient RO permeate. The filamentous yeast strains, *S. clavata* and *M. spicifer*, were initially tested for urease using Christensen’s Urea Broth assay. The principle is based on the presence of urease, which converts urea in a one-step reaction producing ammonia thereby increasing the pH of the broth resulting in a color change. Rapid urease-positive organisms can change the medium’s color to pink within 2–4 h (Kurtzman et al., 2011). Both the filamentous yeast strains tested were negative in this assay. However, when grown in the twice RO-filtrated permeate water, they both metabolized urea and produced ammonia. Both *M. spicifer* and *S. clavata* belong to Hemiascomycetes, which include one class and order (Saccharomycetes and Saccharomycetales). It has been observed that yeasts belonging to Hemiascomycetes contain urea amidolyase, instead of urease (Souciet et al., 2000; Sybren De Hoog and Smith, 2004, 2011a,b; Strope et al., 2011). Urea amidolyase is an enzyme that breaks down urea into ammonia and carbon dioxide through a two-step process. Although a slower mechanism of urea metabolism, it may be advantageous in some settings, since urea amidolyase, in contrast to urease, is not dependent on the concentration of Ni^2+^ and Co^2+^ (Booth and Vishniac, 1987; Navarathna et al., 2010; Strope et al., 2011).

### Heat Tolerance Assay in Saline Peptone Solution

The *D*-values can be affected by several parameters such as strain differences, growth phase and conditions prior to heating as well as the matrix and recovery methods (Doyle et al., 2001) and comparison between different experiments should therefore be done with caution. Nevertheless, if the *D*-values of the *S. clavata* strain (*D*_60°*C*_ = 16.37 min) and the *M. spicifer* (*D*_60°*C*_ = 7.24 min) are compared with previously published average values for common bacterial foodborne pathogens such as *Listeria monocytogenes* (*D*_60°*C*_ = 4.56 min) and *E. coli* O157:H7 (*D*_60°*C*_ = 3.37 min) (Buzrul and Alpas, 2007) in a similar matrix (1% peptone solution), both filamentous yeast strains seem markedly more tolerant to heat treatment. The more heat resistant *S. clavata* has three to four times higher *D*_60°*C*_ than the strains of *L. monocytogenes* and four times or more than the strains of *E. coli* O157:H7 reported by Buzrul and Alpas (2007). Although the two yeasts were very close in taxonomy, morphology and physiology, *S. clavata* was markedly more heat tolerant, indicating that important functional characteristics may vary considerably.

In terms of the CIP programs applied in this type of food industry, cleaning solutions and water flushes are often applied at a temperature of 50°C for 35–45 min for each step (Table 1), since most of the RO membrane elements used in these lines cannot stand higher temperatures (Stoica et al., 2018; Vitzilaiou et al., 2019). Although the CIP cleaning may last more than 2 h, our results indicate that a temperature of 50°C will not ensure inactivation of biofilms containing these yeasts. According to the assay, temperatures of 70°C and higher for more than 10 min ensured at least 4 log reductions. This could explain previously reported observations (Stoica et al., 2018; Vitzilaiou et al., 2019) from some heat tolerant elements being exposed to high heat (78°C/20 min) where no filamentous yeasts were detected.

### UV-C Tolerance

A UV dose of 40 mJ/cm^2^, usually applied in industrial scale water treatment, reduced the population of both *S. clavata* and *M. spicifer* by approx. 4 log_10_ (CFU/mL), but not below the LOD of the method. Moreover, higher doses of 60 and 100 mJ/cm^2^, did not result in significantly higher inactivation, according to one way ANOVA conducted (*P* > 0.001). Bowker et al. (2011), using a similar UV-C LED set-up at 255 nm and phosphate buffer, needed a dose of 9 mJ/cm^2^ to decrease a 10^8^ cells/mL inoculum of *E. coli* by 2.7 log_10_ (CFU/mL). In this study, doses of 20–40 mJ/cm^2^ were needed to obtain the same decrease in the filamentous yeast isolates. UV-C is a well-recognized treatment for water and there is extensive information available for bacteria and viruses as summarized in several reviews (Song et al., 2016; Li et al., 2019). However, there is limited knowledge on fungal tolerance toward UV treatments and the results reported here expands our knowledge of the sensitivity of filamentous yeast.

### Biofilm Tolerance to CIP Assay

The budding yeast strain *S. lactativora* (*SL*) was found in some of the biofilm structures on the RO membrane elements together with the filamentous yeasts, but in lower numbers. When this strain and the filamentous strain, *M. spicifer* (*MS*), were tested for their tolerance toward CIP treatments, by forming biofilms on RO membrane coupons and exposing them to the industrial CIP solutions, they both displayed a considerable tolerance. Combination treatments inactivated more cells than acid or alkaline solutions individually. No difference in survival was found due to the order of application of the acidic and the alkaline solution. In production lines, the chemically dependent effect of the CIP solutions will only be exerted on the retentate side of the RO. According to our previous work examining RO elements from production, filamentous yeasts were detected on both the retentate and the permeate side when CIP treatments applied the acidic solution first, followed by the alkaline while yeasts were only detected on the retentate surface of one RO element in which the alkaline solution was applied first and the acidic at the end (Stoica et al., 2018; Vitzilaiou et al., 2019). Since the inactivating effect of the combinations seems to be similar, it could indicate that the membranes become more prone to hyphae breakthrough when the treatment finishes with alkali and it has been suggested that alkaline solutions can lead to membrane pore expansion (Simon et al., 2013). This merits further investigation.

The biofilm of the budding yeast isolate during the membrane coupon experiments was less affected by the CIP treatment in the laboratory-based experiments. However, the filamentous yeasts found to be the dominant microorganisms on the retentate side of RO membrane elements from industry and the only biofilm former on the RO permeate surfaces after CIP. When the individual cultures of the filamentous and the budding yeast isolate were tested for biofilm formation on polystyrene flat-bottomed microplates with high and low nutrient broth, only the filamentous yeast was able to attach and form biofilm. This was also evident when biofilm formation was assessed on peg lids. The budding yeast biofilm was hardly noticeable and recovery cell numbers after sonication were below detection level. It is likely that the filamentous yeasts may survive shear and chemical stress better due to a tighter adhesion, greater coverage and potential for making mature biofilms and they may use their hyphae to exploit temporarily induced changes of the membranes.

The recent reports on the isolation of *Saprochaete* and *Magnusiomyces* genera from household dishwashers in multi-genera biofilms (Zalar et al., 2011; Döğen et al., 2013; Gümral et al., 2016; Zupančič et al., 2016) confirm their ability to colonize and persist on surfaces exposed to extreme conditions like heat, and periods of desiccation. They also suggest that these yeasts are commonly found in the general environment and it may be speculated that they are spread by a water transmission route. Although they may be common contaminants, *S. clavata* and species belonging to *Magnusiomyces* genera are sometimes referred to as opportunistic pathogens, since they have been associated with nosocomial outbreaks in immunocompromised patients (Gurgui et al., 2011; Birrenbach et al., 2012; Camus et al., 2014; Picard et al., 2014; Vaux et al., 2014; Ulu-Kilic et al., 2015; Del Principe et al., 2016; Favre et al., 2016; Durán Graeff et al., 2017). An association with hospital vacuum flasks was found in one outbreak, but in general, little is known about the occurrence, ecology and routes of transmission.

The data obtained from this study can serve as a new insight to understand the role that filamentous yeast may play in dual or multispecies biofilms and points toward a harboring role of the filamentous yeast species within some biofilm structures, potentially having an impact on the presence and survival of other species. In the specific setting investigated, both the retentate and the permeate were subjected to further treatment downstream before being used and in this environment the role is likely limited to occasional flux problems. However, there could be many membrane processes where these yeasts play a role and it is suggested that future investigations of biofouling should include methods targeting fungal contamination in order to clarify their relevance in different systems.

## Conclusion

Filamentous yeast species identified as the closely related species *S. clavata* and *M. spicifer*, found to dominate biofilms on whey water associated CIP treated RO membrane retentate and permeate surfaces, were characterized by physiological/biochemical tests, stress tolerance assays and biofilm formation potential. These yeasts develop long hyphae and may cover large areas on the RO membranes compared with bacteria and budding yeasts, although they grow much slower than bacteria. Physiological and biochemical tests showed that they share similar colony and cell morphology and biochemical characteristics. The tested isolates of *S. clavata* and *M. spicifer* were found to be tolerant to heat with less than 1 log_10_ (CFU/mL) reductions of *S. clavata* at 60°C after 15 min. Temperatures above 70°C ensured a substantial reduction of both filamentous yeasts. UV-C light at a dose level of 10 mJ/cm^2^ had little effect, while doses of 40 mJ/cm^2^ and upward ensured a 4 log or higher reduction in a static laboratory scale set-up. Both filamentous isolates were able to metabolize urea and grew in the low nutrient twice RO-filtrated permeate.

*Magnusiomyces spicifer* and *S. lactativora* formed robust biofilms on RO membrane coupons that survived sanitizing treatments applied in lab-scale experiments, mimicking industrial CIP treatments. When a filamentous *S. clavata* isolate and a budding yeast *S. lactativora* isolate, both isolated from a RO membrane, were tested for biofilm formation on polystyrene microtiter plates, the filamentous yeast formed a copious biofilm according to 0.1% CV and XTT/menadione staining, as well as on peg lids, while the budding yeast *S. lactativora* isolate did not. When both yeasts were combined, a robust mixed biofilm was formed on peg lids indicating that the filamentous yeast can act as a harbor for the attachment and proliferation of other microorganisms. There has previously been little focus on these filamentous yeasts and the existing sequencing databases reflect the current limitations regarding taxonomy. Their ability, demonstrated here, to attach and proliferate in stressful, nutrient poor environments helps to explain how they persist on membrane surfaces despite regular CIP treatments and is thus a step towards understanding their role in membrane biofouling and flux as well as a potential impact on the quality of retentates and permeates.

## Data Availability Statement

The datasets generated for this study are available on request to the corresponding author.

## Author Contributions

EV conceptualized and designed the work, acquired, analyzed and interpreted the data, wrote and reviewed the manuscript. SA contributed to the initial acquisition of data and review of the manuscript. NM acquired the data and reviewed the manuscript. SK conceptualized the work, supervised the project, interpreted the data and reviewed the manuscript. All authors read and approved the final manuscript.

## Conflict of Interest

The authors declare that the research was conducted in the absence of any commercial or financial relationships that could be construed as a potential conflict of interest.
